# Relation between Skin Pharmacokinetics and Efficacy in AmBisome Treatment of Murine Cutaneous Leishmaniasis

**DOI:** 10.1128/AAC.02009-17

**Published:** 2018-02-23

**Authors:** Gert-Jan Wijnant, Katrien Van Bocxlaer, Vanessa Yardley, Andy Harris, Sudaxshina Murdan, Simon L. Croft

**Affiliations:** a Department of Immunology and Infection, Faculty of Infectious and Tropical Diseases, London School of Hygiene and Tropical Medicine, London, United Kingdom; b Department of Pharmaceutics, UCL School of Pharmacy, London, United Kingdom; c Pharmidex Pharmaceutical Services Ltd., London, United Kingdom

**Keywords:** pharmacokinetics, pharmacodynamics, amphotericin B, cutaneous leishmaniasis

## Abstract

AmBisome (LAmB), a liposomal formulation of amphotericin B (AmB), is a second-line treatment for the parasitic skin disease cutaneous leishmaniasis (CL). Little is known about its tissue distribution and pharmacodynamics to inform clinical use in CL. Here, we compared the skin pharmacokinetics of LAmB with those of the deoxycholate form of AmB (DAmB; trade name Fungizone) in murine models of Leishmania major CL. Drug levels at the target site (the localized lesion) 48 h after single intravenous (i.v.) dosing of the individual AmB formulations (1 mg/kg of body weight) were similar but were 3-fold higher for LAmB than for DAmB on day 10 after multiple administrations (1 mg/kg on days 0, 2, 4, 6, and 8). After single and multiple dosing, intralesional concentrations were 5- and 20-fold, respectively, higher than those in the healthy control skin of the same infected mice. We then evaluated how drug levels in the lesion after LAmB treatment relate to therapeutic outcomes. After five administrations of the drug at 0, 6.25, or 12.5 mg/kg (i.v.), there was a clear correlation between dose level, intralesional AmB concentration, and relative reduction in parasite load and lesion size (*R*^2^ values of >0.9). This study confirms the improved efficacy of the liposomal over the deoxycholate AmB formulation in experimental CL, which is related to higher intralesional drug accumulation.

## INTRODUCTION

Cutaneous leishmaniasis (CL) is a neglected vector-borne tropical disease caused by intracellular protozoan Leishmania parasites. Current estimates suggest 350 million people at risk, 12 million cases per year, and 1 to 1.5 million new cases annually in more than 98 countries, the majority of which occur in Latin America and the Middle East (1). While mortality is limited for the most common form, localized CL, morbidity is serious due to ulceration, disfigurement, and often permanent scarring after healing of the lesion, which are all associated with social stigmatization. More complex and potentially dangerous forms of CL are diffuse (diffuse cutaneous leishmaniasis, or DCL), chronic (leishmaniasis recidivans, or CCL), or destructive to the nasopharyngeal mucosa (mucocutaneous leishmaniasis, or MCL). Current treatments are hampered in their clinical value by toxicity, side effects, variable efficacy, high cost, or invasive administration route. First-line treatment consists of pentavalent antimonials. Second-line chemotherapeutic options include paromomycin, miltefosine, and amphotericin B (AmB). AmB, a macrocyclic polyene antibiotic and important antifungal agent derived from Streptomyces nodosus, is active due to complexation with ergosterol in leishmanial cell membranes, leading to the formation of pores and ultimately pathogen death (2). Due to infusion-related and (nephro)toxicity issues of the classic colloidal dispersion with deoxycholate amphotericin B (DAmB; trade name Fungizone), lipid formulations with an improved tolerability profile and different physicochemical properties were developed, including a phospholipid complex (Abelcet), a dispersion with cholesteryl esters (Amphocil), a multilamellar liposome (Fungisome), and a unilamellar liposome (AmBisome, or LAmB) (3).

No standard dose regimens have been established for LAmB in the treatment of CL, as published data are limited to small case series or individual case reports (4), but clinical success has been achieved with a daily course of 3 mg/kg of body weight for a total dose of 18 to 21 mg/kg. Due to the need for intravenous administration of LAmB and the related risk of systemic adverse effects, it is typically reserved as a 2nd-line treatment for complex CL. This includes patients with (or at risk of) MCL, DCL, or CCL, as well as cases where lesions are large, numerous, potentially disfiguring, unresponsive to earlier therapeutic attempts, and esthetically or practically infeasible to cure locally. General limitations of LAmB include the high price as well as the requirements for cold chain, slow infusion, and hospitalization (5). Despite the relative safety and efficacy of LAmB in CL, fundamental questions about its pharmacology for this disease remain unanswered. Evaluation of pharmacokinetics (PK) and pharmacodynamics (PD) in preclinical models is important to inform optimal clinical use and learn lessons for drug development. A number of studies have looked at the difference in PK and PD properties of AmB formulations in the treatment of invasive fungal pathologies (6–11), but none have done so for CL. Here, we report (i) the single-dose pharmacokinetics of LAmB and DAmB in both healthy and Leishmania major-infected BALB/c mice, (ii) skin distribution after multiple dosing of LAmB and DamB in murine CL, and (iii) the relationship between dose, intralesional AmB concentrations, and response after LAmB treatment at three dose levels.

## RESULTS

### Single-dose plasma and skin PK in healthy and L. major-infected mice.

**FIG 1:**
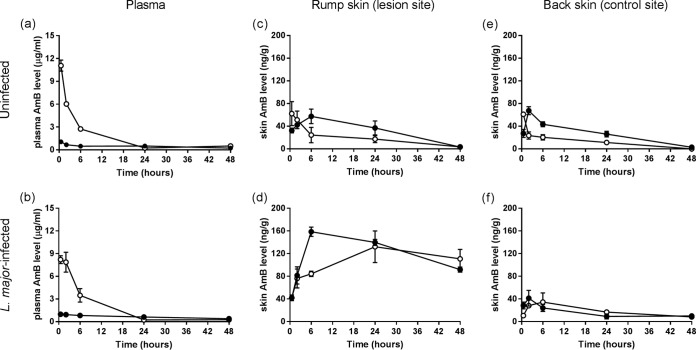
Single-dose pharmacokinetics of the deoxycholate form of AmB (DAmB, •) and AmBisome (LAmB, ○). Uninfected and L. major-infected BALB/c mice received one intravenous dose (1 mg/kg of body weight) of a formulation, after which amphotericin B (AmB) levels in plasma (a and b) and skin at multiple time points were determined. Two skin sites per animal were included: the rump (parasite inoculation site where the localized CL lesion is present in infected [d] but not in uninfected [c] mice) and the back (lesion-free control site in both infected [f] and uninfected [e] animals). Each point represents the means ± SEM (*n* = 4 or 5 per group).

Plasma concentration-versus-time plots after intravenous (i.v.) administration of a single dose of 1 mg/kg LAmB or DAmB to uninfected and L. major-infected mice are shown in Fig. 1a and b, respectively. A dose of 1 mg/kg was used as it is the highest tolerated single dose of DAmB which does not cause signs of acute toxicity (data from pilot studies are not shown). Plasma PK were similar between uninfected and infected mice for the two AmB formulations, with comparable maximum concentration of drug in plasma (*C*_max_), area under the concentration-time curve (AUC), clearance, half-life (*t*_1/2_), and volume of distribution (*V*) (Table 1). However, the plasma profiles for LAmB and DamB individually were significantly different. Compared to DAmB, LAmB achieved a higher plasma peak and systemic exposure (*C*_max_ and AUC around 10- and 3-fold greater, respectively) but showed a shorter half-life and lower clearance and volume of distribution. It should be noted that the terminal phase for LAmB was not clearly defined.

**TABLE 1:** Pharmacokinetic profile of the deoxycholate form of AmB and AmBisome in uninfected and L. major-infected mice after a single i.v. 1-mg/kg dose^*a*^

PK parameter (unit)	Value(s) for:			
	DAmB		LAmB	
	Uninfected	Infected	Uninfected	Infected
*C*_max_ (μg/ml)	1.1	1.0	11.1	8.2
AUC (h · μg/ml)	21.5	30.2	62.7	71.0
Clearance (ml/h/kg)	29.6	18.9	14.2	13.5
*t*_1/2_ (h)	36.1	39.7	10.7	8.5
*V* (ml/kg)	1458	1075	225	143

a Values for pharmacokinetic parameters are calculated from the plasma PK profiles seen in Fig. 1a and b.

Levels of AmB exposure in the rump (lesion site) and back (control site) skin, expressed as the AUC from 0.5 to 48 h (AUC_0.5–48_), are shown in Table 2. In uninfected animals, similar drug distribution profiles in the healthy rump (Fig. 1c) and back (Fig. 1e) tissues were obtained. LAmB gave drug peak levels similar to those of DAmB, around 60 ng/g but at earlier time points (after 30 min versus 2 to 6 h) and only half the total exposure. The rump-to-back AUC_0.5–48_ ratios (1.3 for DAmB and 1.5 for LAmB) indicate that there are limited differences in skin drug exposure based on anatomical location in uninfected mice. In contrast, in L. major-infected animals, the presence of the localized cutaneous lesion on the rump (Fig. 1d) strongly enhanced drug accumulation for both formulations compared to that of the CL-uninfected back skin of the same mice (Fig. 1f). Based on the rump-to-back AUC_0.5–48_ ratios, AmB levels are 6-fold higher for LAmB and 8-fold higher for DAmB. Compared to that of DAmB, LAmB had a similar peak concentration in skin (132 ± 28 versus 159 ± 8 μg/g) at later time points (24 h versus 6 h), showing a trend of slower drug accumulation into and elimination from the lesion. AmB levels in the rump and back tissue for both formulations in infected mice were around 5-fold higher than those in uninfected mice. Changes in AmB plasma concentrations after 1 mg/kg LAmB or DAmB infusion are not reflective for those in skin tissues. No adverse effects at this dose level were observed for either formulation.

**TABLE 2:** Skin distribution of the deoxycholate form of AmB and AmBisome in uninfected and L. major-infected mice after a single i.v. 1-mg/kg dose^*a*^

Skin site	AUC_0.5–48_ value for:			
	DAmB		LAmB	
	Uninfected	Infected	Uninfected	Infected
Rump (lesion site)	1,586 ± 495	6,035 ± 273	863 ± 365	5,270 ± 1,003
Back (control site)	1,269 ± 190	710 ± 194	573 ± 142	915 ± 312
Rump-to-back ratio	1.3	8.5	1.5	5.8

a AUC_0.5–48_ values are calculated from skin PK profiles shown in Fig. 1c, d, e, and f.

### Multiple-dose skin PK and PD in L. major-infected mice.

**FIG 2:**
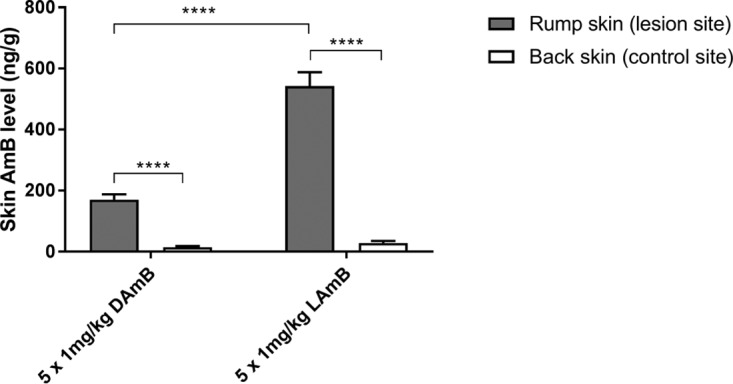
Multiple-dose skin pharmacokinetics of the deoxycholate form of AmB (DAmB) and AmBisome (LAmB). L. major-infected BALB/c mice received intravenous doses of 1 mg/kg on days 0, 2, 4, 6, and 8. On day 10 (48 h after the last dosing), skin samples were collected for amphotericin B (AmB) analysis. The CL lesion was localized on the rump, while the back skin served as a lesion-free, healthy control site. Each point represents the means ± SEM (*n* = 4 to 5 per group). Differences were analyzed using 1-way ANOVA followed by Tukey's multiple-comparison tests and considered significant at a *P* value of <0.05 (*) or not significant (ns) if not (****, *P* < 0.0001).

Skin distribution after multiple dosing of either LAmB or DAmB (1 mg/kg on days 0, 2, 4, 6, and 8) in CL-infected mice is shown in Fig. 2. On day 10, intralesional levels for LAmB (542 ± 46 ng/g) were 3-fold higher than for those for DAmB (170 ± 18 ng/g; *P* < 0.0001). Comparing these concentrations 48 h after the last dosing to those found during earlier single-dose PK studies at the same time point (LamB, 110 ± 17 ng/g; DAmB, 92 ± 4 ng/g) (Fig. 1c and d), a gradual and linear drug accumulation in the target tissue during treatment can be assumed for LAmB but not for DAmB. Again, AmB levels in the lesion were significantly higher than those in the healthy back skin for LAmB (20× higher; *P* < 0.0001) and DAmB (12 higher; *P* < 0.0001).

We then compared the resulting efficacy outcomes for LAmB and DAmB after completing five 1-mg/kg treatments. A small reduction in day 10 lesion size compared to that of the untreated (5% dextrose) group (9.9 ± 0.8 mm) was found for LAmB (9.4 ± 0.2 mm) and DAmB (8.7 ± 0.6), but in both cases the difference was not significant (*P* value of 0.83 and 0.34, respectively). A lower relative parasite load was also found for LAmB (2.0 × 10^7^ ± 0.6 × 10^7^ parasites/g) and DAmB (6.1 × 10^7^ ± 3.4 × 10^7^ parasites/g), but again without a statistically significant difference compared to the control (1.6 × 10^8^ ± 0.5 × 10^8^ parasites/g; *P* value of 0.12 and 0.23, respectively). As expected, both formulations show some antileishmanial efficacy at five treatments of 1 mg/kg, but the toxicity limit of DAmB (1 mg/kg) does not allow a meaningful comparison at clinically relevant dose levels. Because of this, we further investigated only the dose concentration-response relationship at higher doses for LAmB.

### Dose concentration-response of LAmB in L. major-infected mice.

**FIG 3:**
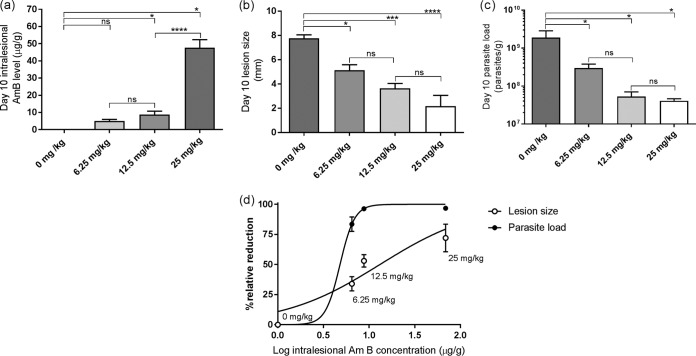
Dose concentration-response relationship of AmBisome (LAmB) in experimental CL. L. major-infected mice received five doses of either 5% dextrose (0 mg/kg; untreated control) or 6.25, 12.5, and 25 mg/kg LAmB (i.v.). On day 10, resulting intralesional amphotericin B levels (a), lesion size (b), and parasite load (c) were evaluated. (d) Outcomes are linked in a logarithmic-scale dose-response curve plotting drug concentrations against relative reduction in lesion size and parasite load. Each point represents the means ± SEM (*n* = 4 to 5 per group). Differences among day 10 outcomes were analyzed using 1-way ANOVA followed by Tukey's multiple-comparison tests and considered significant at *P* values of <0.05 (*), <0.01 (**), <0.001 (***), and <0.0001 (****). ns, not significant.

After L. major-infected mice received 5 doses of LAmB at either 0, 6.25, 12.5, or 25 mg/kg LAmB (on days 0, 2, 4, 6 and 8), the dose level was related to the resulting day 10 intralesional AmB concentrations (Fig. 3a) as well as response, indicated by lesion size and parasite load (Fig. 3b and c, respectively). Figure 3d shows the nonlinear-fit sigmoidal dose-response curve plotting the logarithm of these intralesional AmB levels versus relative reductions in parasite load and lesion size compared to the untreated controls (0 mg/kg). The calculated dose required to achieve 50% (ED_50_) and 90% of maximum effect (ED_90_) was 9.16 and 16.73 mg/kg for lesion size. For parasite load, ED_50_ was 7.55 mg/kg and ED_90_ was 9.16 mg/kg.

We observed a linear dose concentration-response relationship up to 12.5 mg/kg. Between the 0- and 12.5-mg/kg range, correlation was strong between dose concentration (linear regression goodness of fit, *R*^2^ = 0.99) and concentration response (*R*^2^ of 0.99 and 0.91 for relative reduction in parasite load and lesion size, respectively). Little additional efficacy was found by doubling the dose from 12.5 to 25 mg/kg, while intralesional AmB levels increased nonlinearly by 5-fold; this resulted in only a small additional reduction in lesion size and parasite load. This indicates that at 25 mg/kg, the near-maximum efficacy of LAmB for this specific treatment regimen had been reached. Significant reductions in parasite load and lesion size (*P* < 0.05) were found between the control and treated groups at all three dose levels. Doubling of the LAmB dose from 6.25 to 12.5 to 25 mg/kg resulted in a further decrease in parasite load and lesion size, but the differences among the groups were not significant (*P* > 0.05).

## DISCUSSION

The pharmacokinetics and pharmacodynamics of many drugs currently used in the treatment of CL, including different formulations of AmB, are poorly understood (12). We have investigated the single- and multiple-dose skin distribution of AmB following dosing with either the unilamellar liposome AmBisome (LAmB) or the micellar deoxycholate salt form of amphotericin B (DAmB). Significant differences in pharmacokinetics were observed between L. major-infected and uninfected animals, as well as between the two drug formulations.

We observed an important impact of the CL infection on skin accumulation for both LAmB and DAmB. Drug levels in the localized lesion were over 5- to 20-fold elevated compared to those in healthy skin tissue of the same infected mice, as well as in uninfected animals. The pathological condition of CL-infected skin, mainly caused by the severe localized inflammatory immune response against the Leishmania parasites multiplying within dermal macrophages, may explain this phenomenon. After intravenous administration, DAmB dissociates from the colloidal micelles, and over 95% of AmB binds to plasma proteins (13) to form a high-molecular-weight association. LAmB also interacts with proteins, and while 90% of AmB remains stably intercalated in the 60- to 80-nm-sized liposomes (4, 13), coating by opsonins makes the liposomes prone to ingestion by phagocytes in systemic circulation and the reticuloendothelial system in liver and spleen (14). While these complexes have impaired extravasation in healthy skin (continuous endothelium with small vessel pores of 6- to 12-nm diameter [15]), the leaky vasculature at the infection site (increased permeability and disease-inflicted capillary damage) could enhance local drug accumulation (16). Another factor, especially for LAmB, is the migration of phagocytic monocytes, which can serve as potential drug reservoirs, from the bloodstream to the infection site. This is a characteristic of the early-stage and acute immune response against Leishmania (17, 18), causing small, nonulcerated CL nodules (as observed in our L. major-infected mice 12 days postinoculation). Little is known about the elimination of AmB from the target site by local metabolism or lymphatic drainage. However, the latter has been hypothesized as a reason behind the much lower activity of liposomal formulations of AmB (19) and sodium stibogluconate (20) when injected intralesionally compared to intravenously. The impact of these individual physiological processes on local drug distribution in skin is difficult to estimate using the current methodology, which is based on total drug levels and is unable to distinguish between intra- or extracellular, as well as free, protein-bound, or liposome-encapsulated AmB. Furthermore, the general limitations of tissue homogenates apply, such as loss of spatial drug disposition within the compartments of the organ of interest. Novel techniques, such as microdialysis and matrix-assisted laser desorption ionization mass spectrometry imaging, have untapped potential in pharmacological CL research to measure unbound concentrations in the dermal interstitial fluid (21) or study drug disposition within the cellular architecture of infected skin (22). These findings about AmB accumulation in diseased tissue also could be relevant in the treatment of deep cutaneous mycoses (such as invasive candidiasis), where the pathogen, like Leishmania, is located in the dermis (23) instead of the superficial portions of the epidermis where most fungi typically reside.

Comparing the pharmacokinetics of the individual two AmB formulations, we saw significant differences between LAmB and DAmB, consistent with previous studies (24–27). Plasma concentrations and exposure were much higher for LAmB than DAmB and not reflective of changes in skin tissue levels for either formulation. Drug concentrations at the target site were similar after single intravenous dosing of the individual AmB formulations but were 3-fold higher for LAmB than for DAmB following 5-time administration of the same dose. Recently, Iman and colleagues (27) also investigated the distribution of LAmB and DAmB in L. major-infected BALB/c mice, but skin was not evaluated in this study. Increased accumulation of liposomes in inflammatory over healthy sites has also been described for subcutaneous tumors (28), bacterial skin abscesses (29, 30), and fungal infections (31). The so-called enhanced permeation and retention effect, increased drug accumulation at sites of leaky vasculature and defective lymphatic drainage, has been coined as the rationale behind nanoparticle-based drug delivery in cancer and inflammation (16). The data and our understanding of CL histopathology suggest that this effect can also be exploited as a passive targeting strategy in this context by encapsulation of antileishmanial drugs in small (<100 nm), stable (tightly packed phospholipids with cholesterol), unilamellar liposomes (14) similar to AmBisome. Indeed, several promising results have already been achieved with nanoparticles of AmB and other drugs for the treatment of CL (27, 32–37).

Finally, we evaluated how drug concentrations at the infection site after LAmB treatment relate to outcomes. After administration of five consecutive doses, the 1-mg/kg dose of LAmB (as well as DAmB, for which this is the tolerated maximum) proved to be too low to be therapeutic, but a linear dose concentration-response effect was found for 6.25 and 12.5 mg/kg. The clear correlation between intralesional drug levels and treatment outcomes can be explained by the known concentration-dependent manner in which AmB exerts its antimicrobial activity (38). Interestingly, for doubling the LAmB dose from 12.5 to 25 mg/kg, intralesional AmB levels increased by over 5-fold. This could be due to the known phenomenon of saturation of AmB uptake and clearance mechanisms in the organs of the reticuloendothelial system, possibly resulting in higher plasma exposure and increased distribution to other tissues (39). However, little additional efficacy for 25 compared to 12.5 mg/kg was observed. Both of these doses were able to achieve a near-100% reduction in parasite load but not lesion size, indicating the need for longer treatment as the host's response to parasite elimination in the skin is delayed. Results are in line with published data (19, 40) and suggest the clinical superiority of LAmB over DAmB in CL based on enhanced intralesional accumulation of the liposome, as well as already known factors, such as better tolerability and potentially shorter treatment courses. Further PK-PD analysis of LAmB is required to inform optimized clinical dose regimens, especially for the different complex forms of CL, as there are known differences in species-specific drug sensitivity (41), histopathology (17), and immunology (18). It is currently unknown to what degree our observations about skin accumulation of LAmB in the L. major-BALB/c model are translatable to human CL, but understanding of preclinical PK and PK-PD relationships should improve the use and development of antileishmanial drugs.

In summary, intravenous LAmB has potent and dose-dependent *in vivo* activity against CL due to relatively high drug accumulation within the lesion, which is enhanced by the inflamed state of the infected target tissue and the pharmacokinetic properties of the liposomal formulation.

## MATERIALS AND METHODS

### Drugs.

AmBisome (LAmB; Gilead, United Kingdom) and the deoxycholate form of AmB (DAmB; Bristol-Myers Squib, United Kingdom) were reconstituted with sterile water per the manufacturer's instructions to yield stock solutions of 4 mg/ml and 5 mg/ml, respectively. These were diluted in 5% aqueous dextrose to a dose of 1 mg/kg (0.02 mg per dose of 200 μl for mice of a mean weight of 20 g). For LAmB, additional doses of 6.25, 12.5, and 25 mg/kg were similarly prepared. The dilutions were prepared 1 day before starting the experiment and stored at 4°C.

### Parasites.

L. major MHOM/SA85/JISH118 parasites were cultured in Schneider's insect medium (Sigma, United Kingdom) supplemented with 10% heat-inactivated fetal calf serum (HiFCS; Sigma, United Kingdom). These parasites were passaged each week at a 1:10 ratio of the existing culture to fresh medium in 25-ml culture flasks without a filter and incubated at 26°C. For infection of mice, stationary-phase parasites (as confirmed by light microscopy) were centrifuged for 10 min at 2,100 rpm at 4°C. The supernatant was removed and the pellet was resuspended in pure Schneider's insect medium. The number of cells was estimated by microscopic counting with a Neubauer hemocytometer.

### *In vivo* L. major models of CL.

Female BALB/c mice around 6 to 8 weeks old were purchased from Charles River Ltd. (Margate, United Kingdom). These mice were kept in humidity- and temperature-controlled rooms (55 to 65% and 25 to 26°C, respectively) and fed water and rodent food *ad libitum*. After acclimatization for 1 week, mice were randomized and subcutaneously (s.c.) injected in the shaven rump above the tail with 200 μl of a parasite suspension containing 4 × 10^7^ low-passage-number (fewer than 5), stationary-phase L. major promastigotes in RPMI medium. Uninfected mice received a similar but parasite-free injection of 200 μl RPMI medium instead. Twelve days later, when a 4- to 5-mm nonulcerating nodule had formed on the rump of infected animals, mice were allocated to the different experimental groups to ensure comparable lesion sizes.

### Ethics statement.

All animal experiments were conducted under license X20014A54 according to UK Home Office regulations under the Animals (Scientific Procedures) Act 1986 and EC Directive 2010/63/E.

### Single-dose PK study.

Uninfected and L. major-infected BALB/c mice (*n* = 4 to 5 per group) each received LAmB or DAmB at 1 mg/kg of body weight over a 1- to 2-min period by an intravenous bolus (200 μl). Plasma, rump (lesion site), and back (control site) skin samples were collected at 0.5, 2, 6, 24, and 48 h postinfusion.

### Multiple-dose PK and PD study.

L. major-infected BALB/c mice (*n* = 4 to 5 per group) each received LAmB or DAmB at 1 mg/kg or 5% dextrose over a 1- to 2-min period by an intravenous bolus (200 μl) on days 0, 2, 4, 6, and 8. Skin samples from rump (lesion site) and back (control site) were collected on day 10 (48 h after the 5th and final drug administration). This day 10 time point of sacrifice allowed direct comparison with the outcomes of the single-dose PK study (last time point, 48 h). The alternate-day dosing regimen was based on earlier data on the efficacy of LAmB in the L. major-BALB/c model of CL (19). The PD methodology can be found in the following section.

### Dose concentration-response study.

L. major-infected BALB/c mice (*n* = 4 to 5 per group) each received LAmB (i.v.) at 0, 6.25, 12.5, or 25 mg/kg on days 0, 2, 4, 6, and 8. Lesion size was measured daily in two dimensions (length and width) using digital calipers, and the mean size (average of length and width) was calculated. On day 10, rump (lesion site) and back (control site) skin samples were collected and parasite load was evaluated. The methodology to extract parasite DNA from lesions and quantify parasite load by quantitative PCR has already been described in full detail (42).

### Skin sample collection and preparation.

After sacrificing mice (using CO_2_), skin was harvested by surgical removal from the areas containing the localized CL lesion (at the parasite inoculation site on the rump above the tail, termed the lesion site) and non-CL-infected skin on the back (control site). The skin tissue was cut into fine, long pieces and placed into SureLock microcentrifuge tubes (StarLab, United Kingdom) together with 1 spatula (about 100 mg) of 2-mm zirconium oxide beads (Next Advance, United Kingdom) and 1 ml phosphate-buffered saline (PBS; with 0.9% NaOH, pH 7.4; Sigma, United Kingdom). Samples were ground using a Bullet Blender Storm 24 (NextAdvance, United Kingdom) set at speed 12 for 20 min to obtain a smoothly flowing homogenate and stored at −80°C until further use. The homogenate (50 μl) was added to 250 μl of a mixture of 84:16 methanol-dimethyl sulfoxide (DMSO) (high-performance liquid chromatography grade; Fisher Chemical, United Kingdom) containing 200 ng/ml tolbutamide (analytical standard; Sigma, United Kingdom) internal standard for drug extraction and protein precipitation in 96-well plates. Plates were shaken for 10 min at 200 rpm and centrifuged for 15 min at 6,600 rpm at 4°C. One hundred fifty microliters of supernatant was collected and stored at −80°C until analysis. Blanks with and without internal standard as well as calibration samples with known concentrations of AmB (similarly extracted and prepared after spiking 45 μl blank skin homogenate [derived from untreated BALB/c mice] with 5-μl working solutions of known AmB concentrations in 1% SDS [Sigma]) were included.

### Plasma sample collection and preparation.

Blood samples were taken from live animals by needle pricks in the lateral tail veins and collected in Eppendorf tubes preloaded with heparin (2 μl of a 1,000 U/ml stock in sterile water). After centrifugation at 6,500 rpm at 4°C for 10 min, the supernatant plasma was collected in new tubes. Plasma samples for which concentrations of AmB above the upper limit of quantification were expected were first diluted with drug-free blank plasma derived from untreated BALB/c mice. Twenty microliters of plasma was added to 100 μl of a 200-ng/ml tolbutamide internal standard in 84:16 methanol-DMSO. Supernatant (60 μl) was collected and further treated as described for skin samples. Again, blanks with and without internal standard and calibration standards (similarly extracted and prepared after spiking 18 μl blank plasma [derived from untreated BALB/c mice] with 2-μl working solutions of known AmB concentrations in 1% SDS [Sigma]) were included.

### LC-MS/MS quantification of AmB.

The liquid chromatography-tandem mass spectrometry (LC-MS/MS) methodology to quantify AmB levels in experimental leishmaniasis samples was described earlier by Voak et al. (24). Analysis was conducted at Pharmidex Pharmaceutical Sevices, Ltd. (Stevenage, United Kingdom). The lower limit of quantification was 1 ng/ml.

### Pharmacokinetic parameters.

Single-dose PK parameters were estimated assuming noncompartmental analysis in WinNonlin. AUC_0–48_ values for skin were calculated using GraphPad Prism, version 7.02.

### Statistical analysis.

Differences among lesion sizes and parasite loads in the groups were assessed by using one-way analysis of variance (ANOVA) assuming Gaussian distribution followed by Tukey's multiple-comparison test. Data are presented as means and standard errors of the means (SEM). A *P* value of <0.05 was considered statistically significant. All analyses were performed using GraphPad Prism, version 7.02.
